# Insured Patients and the Radcliffe Infirmary

**Published:** 1920-11-20

**Authors:** 


### Insured Patients and the Radcliffe Infirmary.

Mr. H. W. Beesley presided at a recent meeting of the Oxford City Insurance Committee, when it was decided to accede to the request of the Secretary of the Radcliffe Infirmary by raising the rate of maintenance for tuberculous patients in the hospital from 5s. to 8s. a day, as from October 1. It is interesting to note that the great difficulty in obtaining gas prevents all but the most urgent cases from being treated in the dental department.

